# Characteristic pancreatic and splenic immune cell infiltration patterns in mouse acute pancreatitis

**DOI:** 10.1186/s13578-021-00544-1

**Published:** 2021-02-02

**Authors:** Baibing Yang, Joy M. Davis, Thomas H. Gomez, Mamoun Younes, Xiurong Zhao, Qiang Shen, Run Wang, Tien C. Ko, Yanna Cao

**Affiliations:** 1 Department of Surgery, The University of Texas Health Science Center at Houston, Houston, TX 77030 USA; 2 Center of Laboratory Animal Medicine and Care, The University of Texas Health Science Center at Houston, Houston, TX 77030 USA; 3 Department of Pathology & Laboratory Medicine, The University of Texas Health Science Center at Houston, Houston, TX 77030 USA; 4 Present Address: Department of Pathology, George Washington University School of Medicine and Health Sciences, George Washington University Hospital, Washington, DC 20037 USA; 5 Department of Neurology, The University of Texas Health Science Center at Houston, Houston, TX 77030 USA; 6 Department of Genetics, Louisiana State University Health Sciences Center, New Orleans, LA 70112 USA

**Keywords:** Mouse acute pancreatitis, Alcohol, Immune profiling, t-distributed stochastic neighbor embedding (t-SNE), PhenoGraph

## Abstract

**Background:**

A systemic evaluation of immune cell infiltration patterns in experimental acute pancreatitis (AP) is lacking. Using multi-dimensional flow cytometry, this study profiled infiltrating immune cell types in multiple AP mouse models.

**Methods:**

Three AP models were generated in C57BL/6 mice via cerulein (CAE) injection, alcohol and palmitoleic acid (EtOH + POA) injection, and alcohol diet feeding and cerulein (EtOH + CAE) injection. Primary pancreatic cells and splenocytes were prepared, and multi-dimensional flow cytometry was performed and analyzed by manual gating and computerized PhenoGraph, followed by visualization with t-distributed stochastic neighbor embedding (t-SNE).

**Results:**

CAE treatment induced a time-dependent increase of major innate immune cells and a decrease of follicular B cells, and T^CD4+^ cells and the subtypes in the pancreas, whereas elicited a reversed pattern in the spleen. EtOH + POA treatment resulted in weaker effects than CAE treatment. EtOH feeding enhanced CAE-induced amylase secretion, but unexpectedly attenuated CAE-induced immune cell regulation. In comparison with manual gating analysis, computerized analysis demonstrated a remarkable time efficiency and reproducibility on the innate immune cells and B cells.

**Conclusions:**

The reverse pattern of increased innate and decreased adaptive immune cells was consistent in the pancreas in CAE and EtOH + POA treatments. Alcohol feeding opposed the CAE effect on immune cell regulation. Together, the immune profiling approach utilized in this study provides a better understanding of overall immune responses in AP, which may facilitate the identification of intervention windows and new therapeutic strategies. Computerized analysis is superior to manual gating by dramatically reducing analysis time.

## Background

Acute pancreatitis (AP) is an inflammatory disorder associated with substantial morbidity and mortality [1]. Alcohol consumption serves as a major etiologic factor in AP, with alcohol-induced or alcoholic pancreatitis accounting for up to 50% of AP cases [2–4]. While the majority of AP cases resolve without significant sequelae, approximately 16 % progress to chronic pancreatitis (CP), primarily in patients with strong risk factors such as alcohol abuse [5–7]. CP is the number one cause of type 3c diabetes [8] and a major risk factor for pancreatic cancer, one of the most lethal cancer types [9]. Unfortunately, the standard care for pancreatitis lacks specific pharmacological therapies but remains primarily supportive.

The pathogenesis of AP is thought to originate from sterile autodigestion [10], with subsequent inflammatory responses playing a critical role in the disease [11, 12]. Previous studies have shown that infiltrating immune cells, particularly innate immune cells such as neutrophils, macrophages/monocytes, and dendritic cells, are elevated during AP development [13–17], while adaptive or acquired immune cells, including T and B cells, have been reported to be differentially regulated [18–20]. However, previously published works on this topic in experimental AP mouse models used pooled samples from multiple mouse pancreata in order to obtain sufficient cell counts for the measurement of specific immune cells [13, 21]. For these reasons, a comprehensive profiling of the infiltrating immune cells during AP is currently lacking and therefore desired. The outcomes of such a study will lead to a better understanding of the overall immune responses in AP, as well as identify key infiltrating immune cells for the intervention windows and the development of innovative therapeutic strategies, ultimately improving outcomes of AP patients and reducing the risk of developing CP, diabetes, and pancreatic cancer.

For over 50 years, flow cytometry has been used to identify populations of cells including immune cells [22]. Although long-established, this traditional technique has several limitations. For instance, only a few fluorescent-labeled antibodies can be used for detection and the required manual gating analysis is time-consuming with unavoidable biases among individual investigators [23]. To circumvent these limitations, multi-dimensional flow cytometry, with 8–12 fluorescent-labeled antibodies, was developed [24]. Later on, the expansion of computerized analysis led to reduced workload and bias that inherently encompassed the prior manual gating technique, as well as better visualization. For instance, t-stochastic neighbor embedding (t-SNE) emerged, providing a better way to visualize multi-dimensional cytometry data through nonlinear dimensionality reduction according to the similarities of cells, creating an easily understandable 2D or 3D scatter plot [25, 26]. PhenoGraph was then developed with the ability to cluster cells from high dimensional data by converting single-cell data into a graph representing the connections and relationships between the cells, displaying the data on a t-SNE map [27]. Thus, the application of PhenoGraph and t-SNE in multi-dimensional flow cytometry provide powerful tools for a deeper understanding of cell populations and the potential to replace manual gating [23].

In this study, we utilized multi-dimensional flow cytometry to profile infiltrating immune cell types in the pancreas and spleen from three AP mouse models that recapitulate a range of AP pathogenesis. We then analyzed and compared the results by manual gating and computerized analysis. Our study identified characteristic regulation of immune cells in the pancreas and spleen in several experiment AP models, and revealed remarkable time-efficiency of the computerized analysis.

## Results

### Characteristic increase of innate immune cell types but decrease of adaptive immune cell types in the pancreas in cerulein (CAE)-induced acute pancreatitis (AP)

**Fig. 1:**
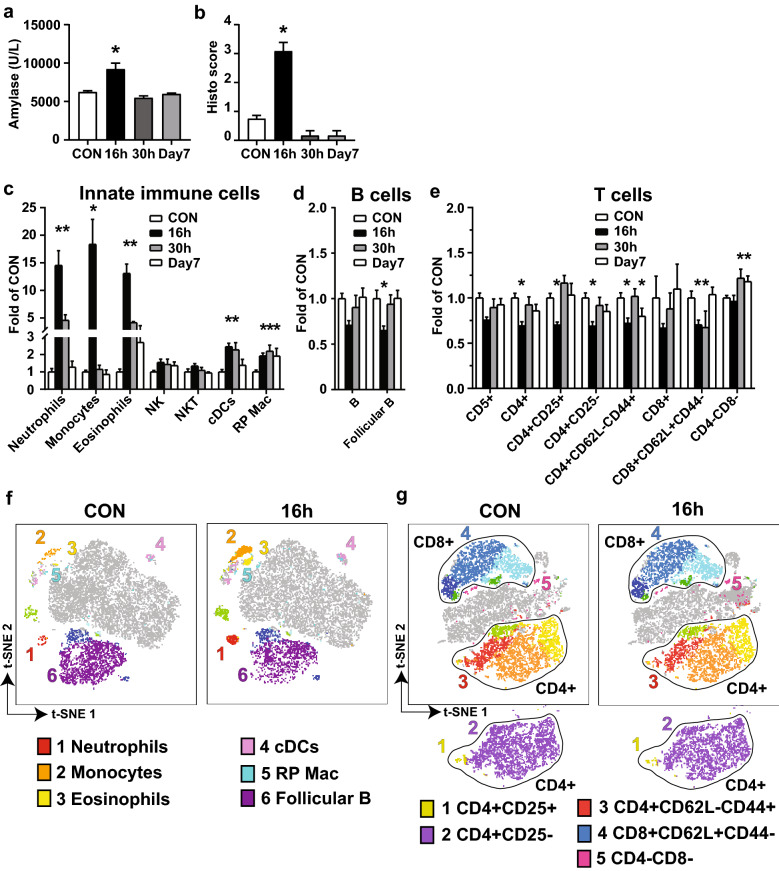
Innate immune cells comprise the majority of infiltrating inflammatory cells in CAE-induced AP pancreas. AP was induced in adult male C57BL/6J mice by CAE injections (50 µg/kg, 9 injections at hourly intervals, ip). Mice were euthanized at 16 h, 30 h, and day 7 after the last CAE injection (n = 6 mice/group). Untreated mice were used as baseline controls (CON. n = 9 mice). The blood and pancreas were harvested. Single cell suspension was prepared from the pancreas. Multi-dimensional flow cytometry was performed using two mouse immune phenotype Panels as described in Table 1, and data was analyzed by manual gating. Results of the cell counts from three separate experiments were normalized against each of the baseline controls, combined and presented as folds of CON. **a** Serum amylase levels. **b** Histopathological scores. **c** Innate immune cells. **d** B cells. **e** T cells. **f** Representative t-SNE showing innate immune cells and B cells measured in Panel 1 on CON and 16 h. **g** Representative t-SNE showing T cells measured in Panel 2 on CON and 16 h. *p < 0.05 compared with CON

To assess the regulation of infiltrating immune cells during AP development, we utilized the widely used and well-defined CAE mouse model in C57BL/6 mice. AP was confirmed by serum amylase level (Fig. 1a) and histopathological scores (Fig. 1b), both increased at 16 h and declined to the control levels at 30 h onward to day 7.

To profile the infiltrating immune cell types in the pancreas during AP development, we isolated pancreatic immune cells from the mice and performed multi-dimensional flow cytometry using two mouse immune phenotype panels (Table 1). By traditional manual gating, we found that the infiltrating immune cells increased during AP development were primarily innate immune cells (Fig. 1c). Compared to the baseline controls (CON), neutrophils (CD11b + Ly6G+) increased by 14.54-fold at 16 h and 4.60-fold at 30 h (p < 0.05), monocytes (or macrophages, CD11b + Ly6C+) increased by 18.36-fold at 16 h (p < 0.05), and eosinophils (CD11b + Ly6G-Ly6C-SSChigh) increased by 13.08-fold at 16 h and 4.16-fold at 30 h (p < 0.05). Conventional dendritic cells (cDCs, IA/IE + CD19-CD11c+) and red pulp macrophages (RP macs, CD11b+/-IA/IE + CD11c-) increased at 16 h (2.45- and 1.92-fold, respectively, p < 0.05) and at 30 h (2.27- and 2.19-fold, respectively, p *<* 0.05). All cell types returned to levels similar to those of CON by day 7, except for RP macs, which were still elevated at day 7.

**Table 1:** Mouse immune phenotype panel 1 and 2

Panel	Marker	Fluor	Dilution	Company	Cat#
1 & 2	CD45	BUV395	1:400	BD (BD Biosciences)	564279
1 & 2	CD5	BV421	1:100	BD	562739
1 & 2	CD161	APC	1:100	BD	550627
1	IA/IE(MHCII)	APC-Cy7	1:100	BioLegend	107628
1	Ly6C	FITC	1:200	BD	553104
1	Ly6G	BV421	1:200	BD	562737
1	CD11b	PE-CF594	1:1600	BD	562287
1	CD11c	PE-Cy7	1:50	BD	558079
1	CD19	BV711	1:100	BD	563157
1	CD21/35	PE	1:200	BD	552957
1	CD23	BV786	1:100	BD	563988
2	CD4	FITC	1:800	BD	553047
2	CD8a	PE-CF594	1:200	BD	562283
2	CD25	PE-Cy7	1:200	BD	552880
2	CD44	PE	1:100	BD	553134
2	CD62L	APC-Cy7	1:100	BD	560514

Regarding the changes in adaptive immune cells, compared to CON, total B cells (IA/IE + CD19+) did not change significantly, but follicular B cells (IA/IE + CD19 + CD23 + CD21/35high) decreased 0.65-fold at 16 h (Fig. 1d, p < 0.05). Total T cells (CD5+) did not change significantly. T^CD4+^, regulatory T^CD4+CD25+^, naïve T^CD4+CD25−^, effector memory T^CD4+CD62L−CD44+^, and naïve T^CD8+CD62L+CD44−^ cells decreased at 16 h (0.69-, 0.70-, 0.69-, 0.62-, and 0.70-fold, respectively, p *<* 0.05), and returned to the levels of CON at 30 h (except for naïve T^CD8+CD62L+CD44−^ cells) and day 7 (except for effector memory T^CD4+CD62L−CD44+^ cells). Double negative T^CD4−CD8−^ cells increased at 30 h and persisted to day 7 (1.22- and 1.18-fold, p < 0.05, Fig. 1e).

To better visualize the multi-dimensional cytometry results, the data from each mouse for each time point were organized by t-SNE, which forms a 2D map showing clusters of the specific cell populations, for the innate immune cells and the adaptive immune cells, B cells, from Panel 1 (Fig. 1f), and for the adaptive immune cells, T cells, from Panel 2 (Fig. 1g).

**Fig. 2:**
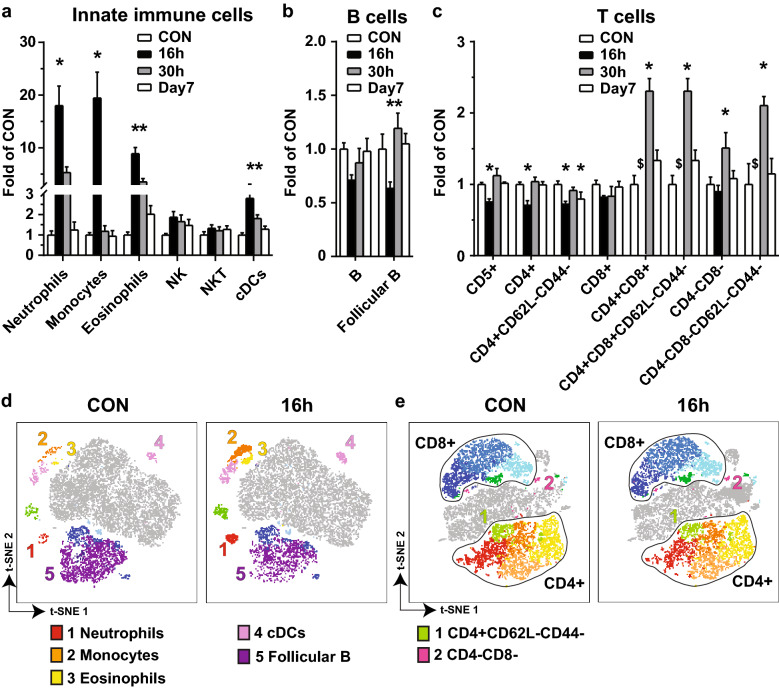
Computerized analysis of the immune cells in CAE-induced AP pancreas. The same sets of flow cytometry data as described in Fig. 1 were analyzed by PhenoGraph. Results of the cell counts from three separate experiments were normalized against each of the baseline controls, combined, and presented as folds of CON. **a** Innate immune cells. **b** B cells. **c** T cells. **d** Representative t-SNE showing innate immune cells and B cells measured in Panel 1 on CON and 16 h. **e** Representative t-SNE showing T cells measured in Panel 2 on CON and 16 h. *p < 0.05 compared with CON. ^$^T subtypes were not clustered by PhenoGraph

We further conducted computerized analysis directly on the CD45 + cells from the multi-dimensional flow data. PhenoGraph was applied and a heatmap was generated based on the surface marker expression. Cell populations were designated according to the combination of the markers. The regulation pattern of the innate immune cells and B cells generated by PhenoGraph was consistent with that by manual gating (Fig. 2a, b). Further, the regulation pattern of T subtypes generated by PhenoGraph was consistent with that by manual gating for T^CD4+^, T^CD8+^ and, T^CD4−CD8−^ cells, but different for total T^CD5+^ cells. The regulation of T^CD4+CD25+^, T^CD4+CD25−^, T^CD4+CD62L−CD44+^, and T^CD8+CD62L+CD44−^ cells was not identified by PhenoGraph analysis. Additional regulated T cell subtypes, T^CD4+CD62L−CD44−^, T^CD4+CD8+^, T^CD4+CD8+CD62L−CD44−^, and T^CD4−CD8−CD62L−CD44−^ cells were identified by PhenoGraph analysis (Fig. 2c). The data were visualized by t-SNE maps from Panel 1 (Fig. 2d) and Panel 2 (Fig. 2e). Overall, the computerized approach took significantly less time and effort that was spent on the manual gating method.

### Differentially regulated innate and adaptive immune cells in the spleen, compared to the immune alterations in the pancreas in CAE-induced AP

**Fig. 3:**
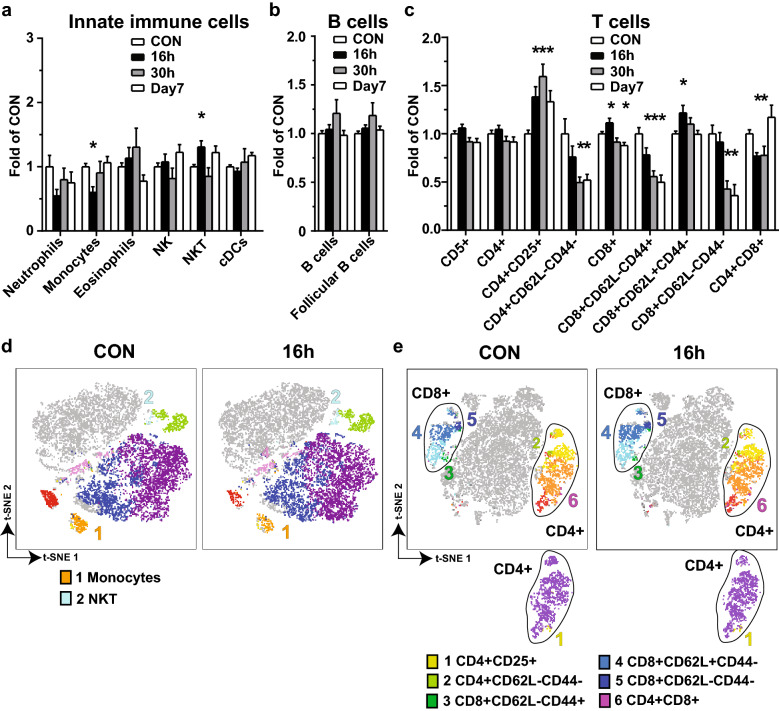
The immune cells are differentially regulated in spleen in CAE-induced AP. Single cell suspension was prepared from the spleen collected from the same sets of the mice as described in Fig. 1. Multi-dimensional flow cytometry was performed using two mouse immune phenotype Panels as described in Table 1, and data was analyzed by manual gating. Results of the cell counts from three separate experiments were normalized against each of the baseline controls, combined and presented as folds of CON. **a** Innate immune cells. **b** B cells. **c** T cells. **d** Representative t-SNE showing innate immune cells and B cells measured in Panel 1 on CON and 16 h. **e** Representative t-SNE showing T cells measured in Panel 2 on CON and 16 h. *p < 0.05 compared with CON

The inflammatory profiles of the spleen were also investigated. Compared to the controls, out of innate immune cells, only monocytes decreased (0.60-fold, p < 0.05), while natural killer T (NKT) cells (CD161 + CD5+) increased at 16 h (1.31-fold, p < 0.05) and returned to the control levels at 30 h and day 7 (Fig. 3a). Regarding the adaptive immune cells, B cells did not change (Fig. 3b). Regulatory T^CD4+CD25+^, T^CD8+^, and naïve T^CD8+CD62L+CD44−^ cells increased, while T^CD4+CD62L−CD44−^, effector memory T^CD8+CD62L−CD44+^, T^CD8+CD62L−CD44−^, and double positive T^CD4+CD8+^ cells decreased (p < 0.05, Fig. 3c). t-SNE was conducted to generate a 2D map for better visualization from Panel 1 (Fig. 3d) and Panel 2 (Fig. 3e).

**Fig. 4:**
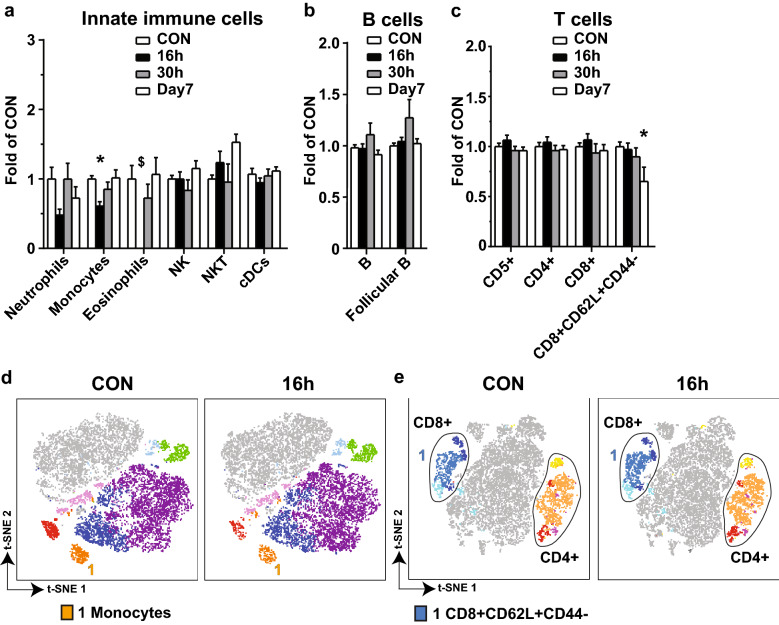
Computerized analysis of the immune cells in spleen in CAE-induced AP. The same sets of flow cytometry data as described in Fig. 3 was analyzed by PhenoGraph. Results of the cell counts from three separate experiments were normalized against each of the baseline controls, combined and presented as folds of CON. **a** Innate immune cells. **b** B cells. **c** T cells. **d** Representative t-SNE showing innate immune cells and B cells measured in Panel 1 on CON and 16 h. **e** Representative t-SNE showing T cells measured in Panel 2 on CON and 16 h. *p < 0.05 compared with CON. ^$^Eosinophils were not clustered by PhenoGraph

Computerized analysis was once again conducted on the CD45 + cells from the multi-dimensional flow data of the spleen, and revealing similar observations for monocytes (Fig. 4a) and B cells (Fig. 4b), but different for regulations on NKT cells (Fig. 4a) and T cell subtypes (Fig. 4c). t-SNE was conducted from Panel 1 (Fig. 4d) and Panel 2 (Fig. 4e).

### Contrasting infiltrating immune cell types in the pancreas and the spleen of alcohol-induced AP models

Alcohol consumption is a major etiologic factor of AP. However, a critical lack of access to human pancreatic tissue has made it difficult to obtain a full understanding of the pathogenic immune mechanisms involved [28]. Therefore, several alcohol animal models have been established, recapitulating human alcoholic pancreatitis for the study of the disease pathogenesis [29–31]. To investigate the regulation of infiltrating inflammatory cells in alcohol-induced AP, we took a similar approach as described in the above-mentioned CAE-induced AP model, and profiled the infiltrating inflammatory cells at 16 h in two alcohol-induced AP models, EtOH + POA and EtOH + CAE.

**Fig. 5:**
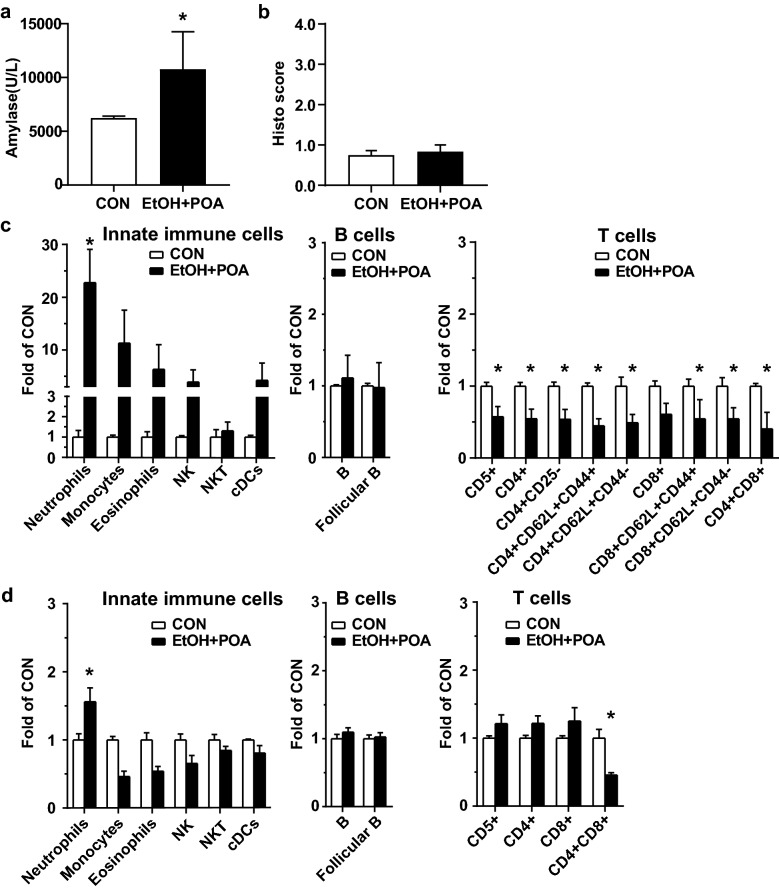
Immune profiles of the pancreas and spleen in the EtOH + POA model. AP was induced in adult male C57BL/6J mice by EtOH and POA injections (EtOH at 1.32 g/kg followed by POA at 4 mg/kg, ip, 1 h apart). Mice were euthanized at 16 h after POA injection (n = 3 mice/group). Untreated mice were used as baseline CON (n = 3 mice). The mouse blood, pancreas, and spleen were harvested for analysis. **a** Serum amylase levels. **b** Histopathological scores. **c** Immune profiles of the pancreas. **d** Immune profiles of the spleen. *p < 0.05 compared with CON

In the EtOH + POA model (Fig. 5), EtOH and POA injections induced higher levels of amylase secretion (Fig. 5a), but similar histopathological scores (Fig. 5b), compared with the controls. Manual gating of the multi-dimensional flow cytometry revealed a similar pattern of innate immune cells and T cells in the pancreas at 16 h, although weaker than that observed in the CAE model (Fig. 5c); neutrophils increased and double positive T^CD4+CD8+^ cells decreased in the spleen (Fig. 5d).

**Fig. 6:**
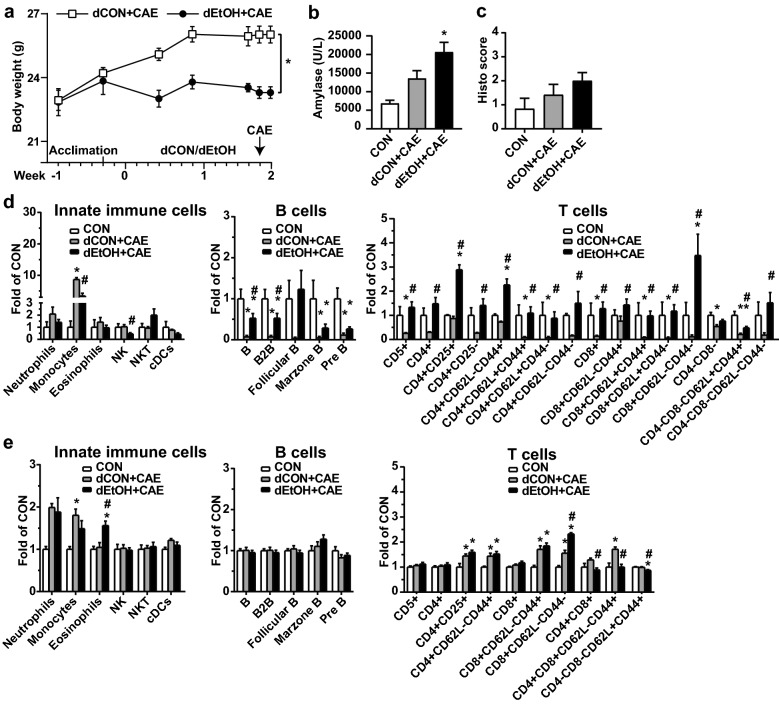
Immune profiles of the pancreas and spleen in the EtOH + CAE model. Adult male C57BL/6J mice were fed with Liebere-DeCarli EtOH liquid diet (5 % v/v EtOH, defined as dEtOH) or pair-fed with control diet (defined as dCON) for 2 weeks and received CAE injections (50 µg/kg, 3 injections at hourly intervals, ip) on the last day. The mice were euthanized at 16 h after the last CAE injection (n = 6 mice/group). Untreated mice were used as baseline CON (n = 3 mice). The mouse blood, pancreas, and spleen were harvested for analysis. **a** Body weight. **b** Serum amylase levels. **c** Histopathological scores. **d** Immune profiles of the pancreas. **e** Immune profiles of the spleen. *p < 0.05 compared with CON. ^#^p < 0.05 compared with dCON + CAE. Marzone B: Marginal zone B cells

In the EtOH + CAE model (Fig. 6), mice fed with EtOH liquid diet (dEtOH) for 2 weeks gained less bodyweight than the mice pair-fed with control diet (dCON, Fig. 6a). CAE injections (3 hourly) combined with EtOH feeding induced higher amylase secretion compared to the baseline controls (CON) and dCON feeding groups (Fig. 6b, *p < 0.05), suggesting that EtOH feeding may synergize the CAE effect on amylase secretion. However, the increase of histopathological scores observed for the combined CAE injection and EtOH feeding group did not reach significant levels (Fig. 6c). In the pancreas, manual gating of the multi-dimensional flow cytometry demonstrated increased monocytes, decreased B cells, and decreased T cells and their respective subtypes in the dCON + CAE group, the effect of which was attenuated in the dEtOH + CAE group (Fig. 6d). In the spleen, increased eosinophils were observed in the dEtOH + CAE group. Increased monocytes and increased regulatory T^CD4+CD25+^, effector memory T^CD4+CD62L−CD44+^, effector memory T^CD8+CD62L−CD44+^, T^CD8+CD62L−CD44−^, and T^CD4+CD8+CD62L−CD44+^ cells were observed in the dCON + CAE group, the effect of which was attenuated in the dEtOH + CAE group, except for T^CD8+CD62L−CD44−^ cells (Fig. 6e).

## Discussion

In this study, we demonstrated a complex and variable regulation of innate and adaptive immune cells in the pancreas and spleen during AP progression. Specifically, we found that in the pancreas, several innate immune cells increased during the acute phase (16 h) and then declined to baseline levels at recovery phases (30 h–day 7), while adaptive immune cells decreased during the acute phase and recovered to baseline levels subsequently. In the spleen, different patterns were observed compared to those in the pancreas, in that most innate immune cells populations did not change in response to AP induction, particularly T cell subtypes were differentially regulated.

Previous studies investigating the immune cell regulation in AP report individual or a limited number of immune cell types independently in human pancreas studies [14, 19], animal CAE model [13, 16, 32] and other animal models [16–18, 20, 33]. Regarding innate immune cell types in literature, neutrophils have been reported to increase in the early phase of AP [32], while monocytes/macrophages increase in the early and late phases of AP [13, 14, 17, 33]. Increased eosinophils have been detected in human eosinophilic pancreatitis [34, 35], the CAE-induced CP model [36], and the partial duct ligation mouse AP model [17]. DCs have been shown to increase after AP induction [16, 17]. Our data, consistently and systematically, demonstrated the increase of neutrophils, monocytes/macrophages, eosinophils, and cDCs at the early phase of AP and the recovery at day 7 in the CAE-AP model (Fig. 1a). NK cells and NKT cells were reported to be increased in the pancreas in adenovirus-mediated mouse AP [33], however we did not observe an increase of NK cells and NKT cells in the pancreas. This variance could be related to pancreatitis induction methods. RP macs are named due to being principally localized in the red pulp of the spleen. They are necessary for maintaining blood homeostasis by phagocytosis of injured and senescent erythrocytes and blood-borne particulates [37]. However, the corresponding population of RP macs in the pancreas is unclear. Here we report an increase of RP macs in the pancreas during AP. Therefore, further study on this special population of cells in the pancreas is warranted. Regarding adaptive immune cells, we observed decreased B cell subtypes in AP, consistent with literature reported in human pancreas study [19]. Variable regulation patterns of T cells and the subtypes have been documented in human pancreas study [14, 19] and animal models [16, 33]. We observed a trend of decreased T cells and the subtypes in AP, and also identified the regulation of several additional T cell subtypes. Overall, our data revealed that the immune cell regulation persisted after serum amylase levels and histopathological changes of the pancreas recovered to baseline levels.

In the spleen, we observed decreased monocytes and increased NKT cells at 16 h, consistent with literature reports [20, 33]. Furthermore, increased T^CD4+^ cells were reported in the blood, liver, and spleen of sodium-taurocholate-induced necrotizing acute pancreatitis in mice [18]. We did not observe increased T^CD4+^ cells, but demonstrated differentially regulated T cell subtypes in the spleen of the CAE-induced AP model.

Multi-dimensional flow cytometry serves as an effective systems biology tool to adequately evaluate complex cellular systems and clarify cellular identity and function [38]. Although supplying a more robust analysis with increased fluorescent-labeled antibody capabilities, the manual gating analysis can be quite taxing on the individual researcher and is inherently prone to bias. Newly developed computerized analysis techniques, such as PhenoGraph and t-SNE, dramatically reduce the time to analyze data while providing parallel, if not greater, quality. Our study demonstrated that computerized analysis techniques took a fraction of the time for analysis and achieved comparable results for the identification of innate immune cells and B cells as that by manual gating. However, further optimization on analyzing T cell subtypes is warranted.

Comparing computerized analysis with manual gating in this study, our study supports the use of computerized analysis techniques directly without manual gating in further studies for innate immune cells and B cells. As high-dimensional flow cytometry (18 to 30 parameters) and mass cytometry (> 40 parameters) are the future direction in the field of immunology research [39, 40], for instance, combining the two mouse immune panels used in the current study would further improve efficiency.

Since there are fewer studies on the infiltrating immune cells in alcohol-induced AP animal models, we performed immune profiling in two alcohol-induced AP models to explore this aspect. We observed a similar immune profile pattern although with a weaker effect, in the pancreas of the EtOH + POA model compared to the CAE model. In the EtOH + CAE model, we only observed increased monocytes out of the tested innate immune cells and decreased B and T cell subtypes in the pancreas in the dCON + CAE group. Interestingly, most of the effects were attenuated in the dEtOH + CAE group (Fig. 6). The opposing effects of EtOH feeding on CAE-induced immune responses appear to be contradicting to its enhancing effect on CAE-induced amylase secretion. Further investigation is warranted to uncover the underlying mechanism.

There are several aspects of novelty in this study. First, we utilized three AP models to recapitulate a range of AP pathogenesis in order to demonstrate a “big picture” of immune cell infiltration and regulation patterns in the pancreas and spleen. Second, we demonstrated a systemic analysis of the immune cell regulation in AP. Third, we discovered an unexpected opposing effect of EtOH on CAE-induced immune cell regulation. In addition, we validated the time efficiency and data reproducibility using computerized analysis by directly comparing with traditional manual gating analysis. There are several limitations in this study. For feasibility reasons, only pancreas and spleen were analyzed in current studies. Peripheral blood and other co-morbid organs should be included, as they have been independently studied in clinical research and animal studies. The alcohol models utilized in this study only induced amylase secretion, with no significant pancreatic histopathological damages, resulting in weaker immune responses than that observed in the CAE model. Therefore, alcohol-induced animal AP models should be optimized to achieve stronger AP induction. Although certain inconsistent patterns of the immune cell regulation were derived from three different AP animal models, upregulation of innate immune cells and downregulation of adaptive immune cells in the pancreas are consistent across these different models, and, importantly, validate the findings derived from clinical studies. Although reproducibility of each experiment is fair, it is still necessary to include baseline controls for each repeat experiment in order to normalize and combine the results within the same treatment. Use of untreated mice as baseline controls is an acceptable option in the pancreas research field, particularly in comparison of multiple time points post-injury [41]. Taking a similar approach, in this study, untreated mice in individual experiments were set as baseline controls in an effort to compare the time-dependent responses post-AP induction, also, to keep consistency to compare magnitude of the responses among different AP models.

## Conclusions

In conclusion, by using multi-dimension flow cytometry, we profiled infiltrating immune cell types in the pancreas and spleen of CAE- and alcohol-induced AP mouse models. Specifically, we found that the majority of infiltrating immune cells in the AP pancreas were innate immune cells, accompanied with downregulated adaptive immune cells. We observed differential regulation patterns in the spleen, where monocyte/macrophages decreased and NKT cells increased, associated with differentially regulated T cell subtypes. The EtOH models demonstrated similar but weaker trends on infiltrating immune cell types when compared to the CAE model. The results in this study further attested that computerized analysis techniques are superior to manual gating in ease and time efficiency, and data reproducibility particularly for innate immune cells and B cells. Overall, the results from this comprehensive profiling demonstrate the time-dependent immune responses in AP, which may lead to identification of cellular targets and time windows for further studies and therapeutic advancements.

## Methods

### Reagents

Cerulein (CAE), the decapeptide analog of the potent pancreatic secretagogue cholecystokinin, was purchased from Bachem Americas, Inc. (Torrance, CA); Phadebas Amylase Test from Magle Life Sciences (Cambridge, MA); alcohol (EtOH, 200 proof, 100%) from Fisher Scientific (Hampton, NH); palmitoleic acid (POA, a non-oxidative ethanol metabolite) from Sigma-Aldrich (St. Louis, MO); and rodent liquid diets from Bio-Serv (Flemington, NJ, Catalog # F1258SP for EtOH feeding, Catalog # F1259SP for pair-fed control).

### Animals and AP induction

All animal experiments were performed according to the protocols approved by the Animal Welfare Committee of the University of Texas Health Science Center at Houston. C57BL/6 mice (6–8 weeks old, male) were purchased from Jackson Laboratories, Inc. (Bar Harbor, ME). The animals were housed in a climate-controlled room with an ambient temperature of 23 °C and a 12:12-h light-dark cycle. Animals were fed standard laboratory chow (LabDiet 5053, LabDiet, St. Louis, MO), given water ad libitum, and randomly assigned to control or experimental groups.

Three AP mouse models were generated: (1) CAE model. AP was induced by CAE (50 µg/kg, 9 injections at hourly intervals, intraperitoneal (ip)) as described previously [42]. The mice were euthanized at 16 h, 30 h and day 7 after the last CAE injection (n = 6 mice/group). (2) EtOH-POA model. The mice were injected (ip) with EtOH (1.32 g/kg) followed by POA (4 mg/kg) 1 h apart [29, 30]. The mice were euthanized 16 h after the POA injection (n = 3 mice/group). (3) EtOH + CAE model. The mice were fed with Liebere-DeCarli EtOH liquid diet (5 % v/v EtOH) or pair-fed with control diet for 2 weeks [31]. All mice received CAE (50 µg/kg, 3 injections at hourly intervals, ip) on the last day. The mice were euthanized 16 h after the last CAE injection (n = 6 mice/group). The mouse blood, pancreas, and spleen were harvested for analysis. For each set of experiment, untreated mice were included as baseline controls (n = 3 mice) to keep consistency for comparison between different sets of experiments.

### Amylase measurement

Serum amylase levels were determined using the Phadebeas Amylase test tablets as instructed by the manufacturer and as previously described [42].

### Pancreatic morphological evaluation

Paraffin-embedded pancreas samples were sectioned (5 µm), stained with hematoxylin & eosin (H&E), and examined by an experienced pathologist blinded to the sample identity. Histopathological changes of AP were evaluated according to the criteria from previously published works [42, 43], and scored from 0 to 4 as absence to the most severe injury on the extent of edema, inflammation, and acinar necrosis.

### Cell isolation

Single cell suspension from the pancreas was prepared as previously described [13, 21]. Briefly, pancreas tissue was minced and digested in digestion buffer (Hank’s balanced salt solution (HBSS) without Mg^2+^, without Ca^2+^, containing 8 % fetal bovine serum (FBS), 2 mg/ml collagenase IV, 20 µg/ml DNase I) at 37 °C and shaking for 15 min. The digested tissue was filtered through a 100 µm cell strainer and washed with fluorescence-activated cell sorter (FACS) buffer (HBSS containing 2 % FBS and 4 % bovine serum albumin). The suspended cells were centrifuged at 400*g* for 5 min. The cell pellet was treated with red blood cell (RBC) lysis buffer and EasyStep dead cell removal kit (StemCell Technologies, Vancouver, Canada), centrifuged at 400*g* for 5 min, and suspended for flow cytometry.

Single cell suspension from the spleen was prepared by the cytometry and cell sorting core (CCSC) of Baylor College of Medicine (Houston, TX). Briefly, spleen tissue was dissociated and digested in digestion buffer (RPMI 1640 containing 2 mg/ml DNase I, 10 mg/ml collagenase II) in the tubes, placed on gentleMACS Dissociator (Miltenyi Biotec, Bergisch Gladbach, Germany), and incubated at room temperature for 10 min, twice. The stopping buffer (1x PBS, 0.1 M Ethylenediaminetetraacetic acid (EDTA)) was added to the digestion mix, which was then filtered through a mesh filter cap and centrifuged at 400*g* for 5 min. The cell pellet was suspended in FACs buffer (1x phosphate-buffered saline (PBS) without Mg^2+^, without Ca^2+^, 2 % FBS, 25 mM HEPES, 2 mM EDTA) and incubated with RBC Lysis buffer, washed again and suspended in FACS buffer for flow cytometry.

### Flow cytometry assays

All flow cytometry assays were performed by CCSC of Baylor College of Medicine. Briefly, the isolated cells were incubated with the block solution (FACS buffer with 1:125 dilution of CD16/CD32), and then stained with the mouse immune phenotype Panel 1 or 2 antibody cocktail (Table 1) at 4 °C for 20 min in dark. The cells isolated from the spleen were also used as the Fluorescence Minus One (FMO) control. The cells were centrifuged at 400*g* for 5 min, and the cell pellet was resuspended in running buffer (FACS Buffer with one drop/ml Nuc Blue Fixed DAPI Stain. Life Technologies, Carlsbad, CA). The flow cytometry reaction was run on LSRII Cell Analyzers (BD Biosciences, San Jose, CA); 500,000 live singlet events for Panel 1 and 350,000 live singlet events for Panel 2 were recorded.

### Data analysis

Cytometry data were analyzed with Flowjo 10 (Treestar Inc, Ashland, OR). All FCS files were cleaned by Flowjo plugin FlowAI [44]. Conventional manual gating was first conducted for identification of cell populations and statistical analysis. Each cell type/population with mean event number more than 100 in any group were adopted to have coefficient of variation ≥ 10 % [45] and analyzed as % of total leukocytes (CD45+). The results of manual gating were visualized in 2D t-SNE maps. Briefly, the CD45 + cells of all samples were downsampled to 3000 (or all for events less than 3000) and concatenated. t-SNE was run on the global concatenated data with iteration of 1000, perplexity of 30, learning rate of 200 and theta of 0.5 to obtain a 2D map by flowjo plugin t-SNE [46]. Group gating by sample ID was conducted on the global concatenated data to obtain group concatenated data as well as t-SNE maps for each group. Cell populations of each group were superposed on t-SNE maps respectively. For computerized analysis, PhenoGraph was run on the concatenated data with the parameter k = 30 to define subpopulations by Flowjo plugin PhenoGraph [27], and the clusters generated by PhenoGraph were then applied to each group and superposed onto t-SNE maps. A heatmap of surface marker expression was generated by Flowjo plugin ClusterExplorer for interpretation of the cell populations clustered by PhenoGraph.

### Statistical analysis

Statistics were conducted with GraphPad Prism 8 (GraphPad Software. La Jolla, CA) and SPSS 24.0 (IBM Corp., New York, NY). Data are presented as mean ± standard error (SE). One-way ANOVA followed by LSD post hoc analysis was used to determine the differences among multiple groups. Student’s t test was used to compare the difference between two groups. p value less than 0.05 was considered significant.

## Data Availability

All data generated or analyzed during this study are included in this article.

## Abbreviations

AP: Acute pancreatitis
CP: Chronic pancreatitis
CAE: Cerulein
EtOH: Alcohol
POA: Palmitoleic acid
t-SNE: t-distributed stochastic neighbor embedding
NK cells: Natural killer cells
cDCs: Conventional dendritic cells
RP macs: Red pulp macrophages
